# A tailored graphene supramolecular gel for pharmaceutical crystallization†

**DOI:** 10.1039/d4sc08087d

**Published:** 2025-03-24

**Authors:** Qi Zhang, Martin A. Screen, Leon Bowen, Yisheng Xu, Xiangyang Zhang, Jonathan W. Steed

**Affiliations:** a State Key Laboratory of Chemical Engineering, East China University of Science and Technology Shanghai 200237 China zxydcom@ecust.edu.cn jon.steed@durham.ac.uk; b Department of Chemistry, Durham University Durham DH1 3LE UK; c Department of Physics, Durham University Durham DH1 3LE UK

## Abstract

A graphene-based supramolecular gel was designed and prepared to control the crystallization process and polymorphism of pharmaceuticals. The gelators were modified at the end segments with pyrene moieties, which spontaneously interact with the graphene surface by aromatic stacking interaction resulting in a graphene-incorporated supramolecular gel linked by noncovalent interactions between urea groups. When graphene was included into the gel, the critical gel concentration and system rigidity changed significantly, fluorescence spectroscopy determined the close π–π stacking interaction between the gelator and graphene, and the material was confirmed as a true nanocomposite gel system by electron microscopy. Further the graphene was oxidatively modified to obtain hydroxylated graphene (Gr–OH), which was successfully incorporated into the gel system to serve as a medium for pharmaceutical crystallization. Glycine (GLY), caffeine (CAF) and aripiprazole (APZ) were selected as model drugs for gel surface crystallization and gel phase crystallization by Gr–OH hybrid gels. Incorporation of Gr–OH in the gel allowed close interaction by hydrogen bonding with drug molecules, resulting in different polymorphs of GLY, CAF and APZ compared to solution crystallization and shorter induction time of CAF compared to the native gel.

## Abstract 2

A graphene-based supramolecular gel was designed and prepared to control the crystallization process and polymorphism of pharmaceuticals.

## Graphical abstract

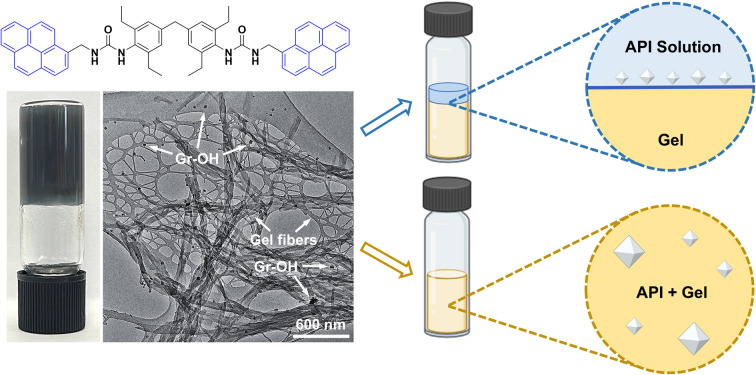

## Introduction

Controlling the crystallization of solid pharmaceuticals is crucial for drug properties.^1^ The polymorphism, crystal habit, and nucleation and growth behavior of active pharmaceutical ingredients (APIs) are influenced by the crystallization process,^2^ and the final solid form can affect the stability, solubility, dissolution rate, and bioavailability of APIs,^3^ as well as the flow and compaction properties in formulations.^4^ Emerging heterogeneous nucleation methods are effective tools to counter the challenges of crystallization control in traditional solution crystallization processes,^5^ including graphene template crystallization,^6,7^ gel phase crystallization,^8,9^ and nanoconfinement crystallization.^10–13^ Each of these methods has specific advantages in discovering new polymorphs, controlling morphology, obtaining high-quality single crystals, and understanding the crystallization mechanism.^14^

Graphene (Gr), is a well-known two-dimensional nanomaterial,^15^ and its surface properties are easily modified by etching and covalent functionalization,^16,17^ making it a promising substrate for crystallization control.^6^ Initially used as a template for the preparation of inorganic materials and polymers,^18,19^ graphene has recently begun to be used for the crystallization of organics and pharmaceuticals.^20,21^ Specific intermolecular interactions can be identified by the surface chemistry of graphene to induce particular polymorphs of APIs by additive-templated and substrate-templated crystallization methods.^22^ However, the low dispersion concentration of graphene in solution and the interface between solid and liquid may prevent it from interacting closely enough with drug molecules, limiting its effect on the crystallization process. Incorporation of graphene within a gel medium offers a potential approach to increasing graphene effective concentration and enhancing its influence through nanoconfinement within the gel matrix.

Supramolecular gels are viscoelastic materials that can act as effective crystallization media.^23,24^ A series of gelators have been developed for the crystallization of various APIs,^25^ such as carbamazepine,^9,26–29^ ROY,^29,30^ sulfathiazole,^31,32^ thalidomide,^33^ metronidazole,^34^ and mexiletine hydrochloride,^35^ in which the solvent trapped in the gel network can act as a confined crystallization medium to control the specific nucleation and growth process of drug molecules within the gel.^8,36^ In addition, the supramolecular gel network structure can provide porous pockets to accommodate various nanomaterial hosts by physical hybridization,^37–40^ and some gelators can interact chemically with nanomaterial to form the incorporated gel system.^41–43^ Therefore, it should be possible to incorporate graphene into the gel as a soft template for crystallization control, allowing sufficiently close interaction between the graphene surface and drug molecules.

**Scheme 1:**
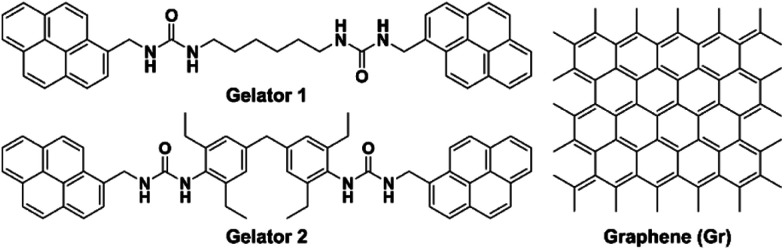
Chemical structure of gelators 1 and 2 containing bis-pyrene moieties and graphene.

In this work, two gelators containing pyrenyl moieties were synthesized (Scheme 1), of which gelator 2 proved to be an effective gelator and was chosen for subsequent studies. Graphene was successfully incorporated into the gel system through π–π stacking with the pyrenyl groups. To achieve stronger interaction with the API and hence influence the API crystallization outcome, the graphene was further functionalized by hydroxylation (Gr–OH), which also facilitated incorporation into the gel. Finally, the Gr–OH gel was used to explore the effect on polymorphism and nucleation behavior of glycine (GLY), caffeine (CAF), and aripiprazole (APZ) by gel surface crystallization and gel phase crystallization.

## Results and discussion

### Screening and characterization of pyrene-containing gel

Both gelators 1 and 2 were synthesized in very good yield by a nucleophilic addition reaction in chloroform of 1-pyrenylmethanamine hydrochloride with the corresponding terminal isocyanate-containing linker at a 2 : 1 ratio, as described in the experimental section. The two low-molecular-weight gelators both contain two urea groups, which form supramolecular gels primarily through intermolecular hydrogen bonding interactions. The supramolecular gels are readily prepared by simply dissolving the gelator in a solvent by heating and either standing or slightly sonicating at room temperature.

**Fig. 1:**
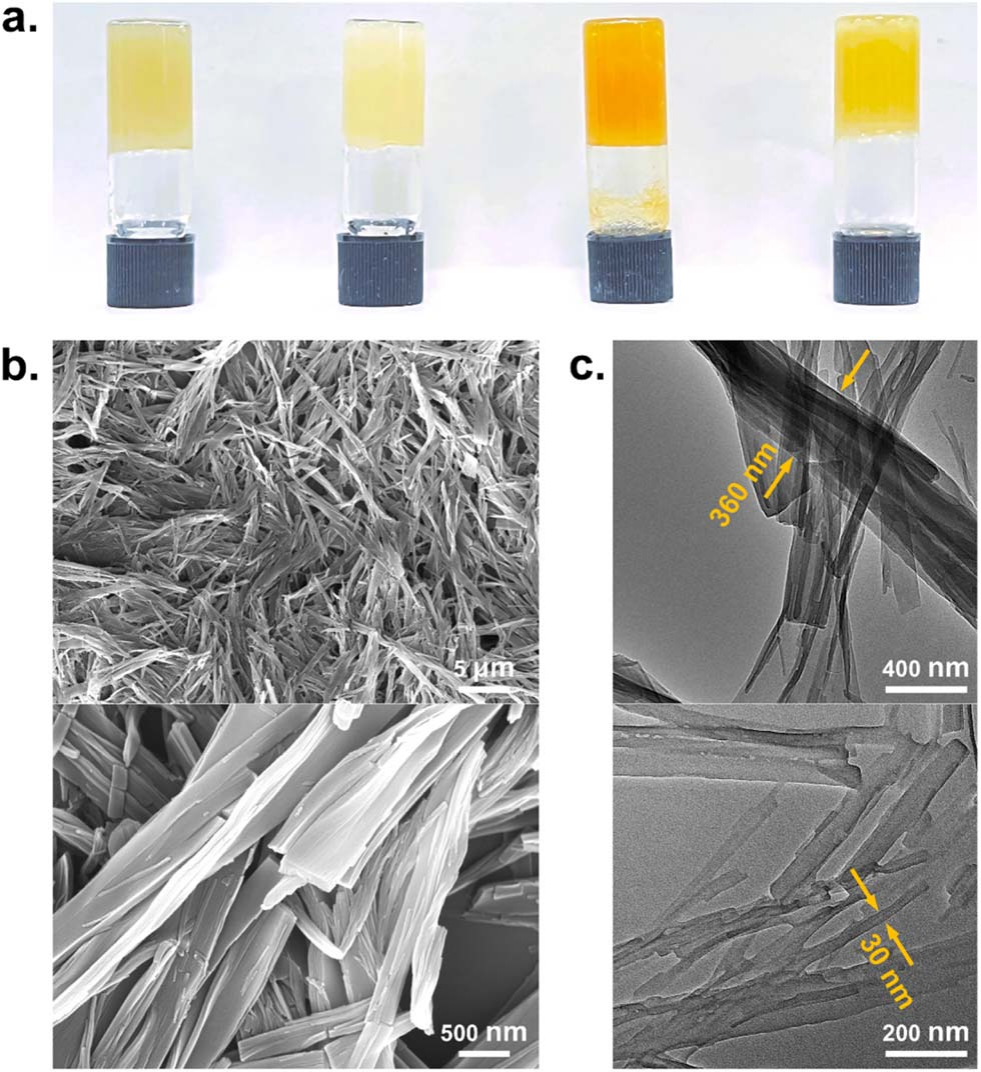
(a) Gels at CGC formed by gelator 2 in (left to right) 1,2-dichlorobenzene, DMSO, 1,2,4-trichlorobenzene and benzyl alcohol; (b) SEM and (c) TEM images of the DMSO gel of gelator 2 at 0.85% w/v.

Gel screening was carried out for both gelators. Despite many solvent candidates, the gelators showed solubility and gelation in only a few solvents due to the presence of pyrene groups which give rise to relatively low solubility (Table 1). Gelator 1 is less soluble than 2, and gelation occurred only in benzyl alcohol and dimethyl sulfoxide (DMSO). Gelator 2 demonstrated excellent gelation ability, forming gels at low critical gel concentration (CGC). Fig. 1a shows the gel formation of 2 in 1,2-dichlorobenzene, DMSO, 1,2,4-trichlorobenzene and benzyl alcohol at the CGC values of 0.6, 0.85, 1.0, and 1.5% w/v, respectively. Considering the incorporation of graphene into the gel and its application in drug crystallization, we selected the gels formed by 2 in DMSO for further studies, since 1,2-dichlorobenzene requires heating to its boiling point (about 150 °C) before significant dissolution of 2 occurs. Moreover, most APIs are soluble in DMSO.

**Table 1:** Gelation behaviors of 2% w/v gelators 1 and 2 in different solvents

Solvent	Gelator 1^a^	Gelator 2
1,2-Dichlorobenzene	I	G
1,2,4-Trichlorobenzene	S	G
Benzyl alcohol	G	G
Dimethyl sulfoxide	PG	G
*N*,*N*-Dimethylformamide	I	S
Ethylene glycol	S	S
Butane-1,4-diol	I	S
3-Chloro-1-propanol	S	S

a I = insoluble with heating, S = solution, G = gel, and PG = partial gel.

Morphologies of the DMSO xerogel of 2 were characterized by scanning electron microscopy (SEM) and transmission electron microscopy (TEM). SEM images reveal the entangled nanofibrillar structure of the gel, and these gel fibers are highly cross-linked, shown in Fig. 1b. TEM images show the width of the fibers as 30–360 nm and the length of each fiber as more than 1 μm, shown in Fig. 1c. The width of the gel fiber is much higher than the molecular size of a gelator, suggesting that multiple molecular chains self-assemble to form supramolecular fibers, possibly *via* a scrolling mechanism.^44,45^ The formation of entangled fibers demonstrates that the pyrene-containing gelators form the supramolecular gel network as cross-linked aggregates.

### Incorporation of graphene into the gel

**Fig. 2:**
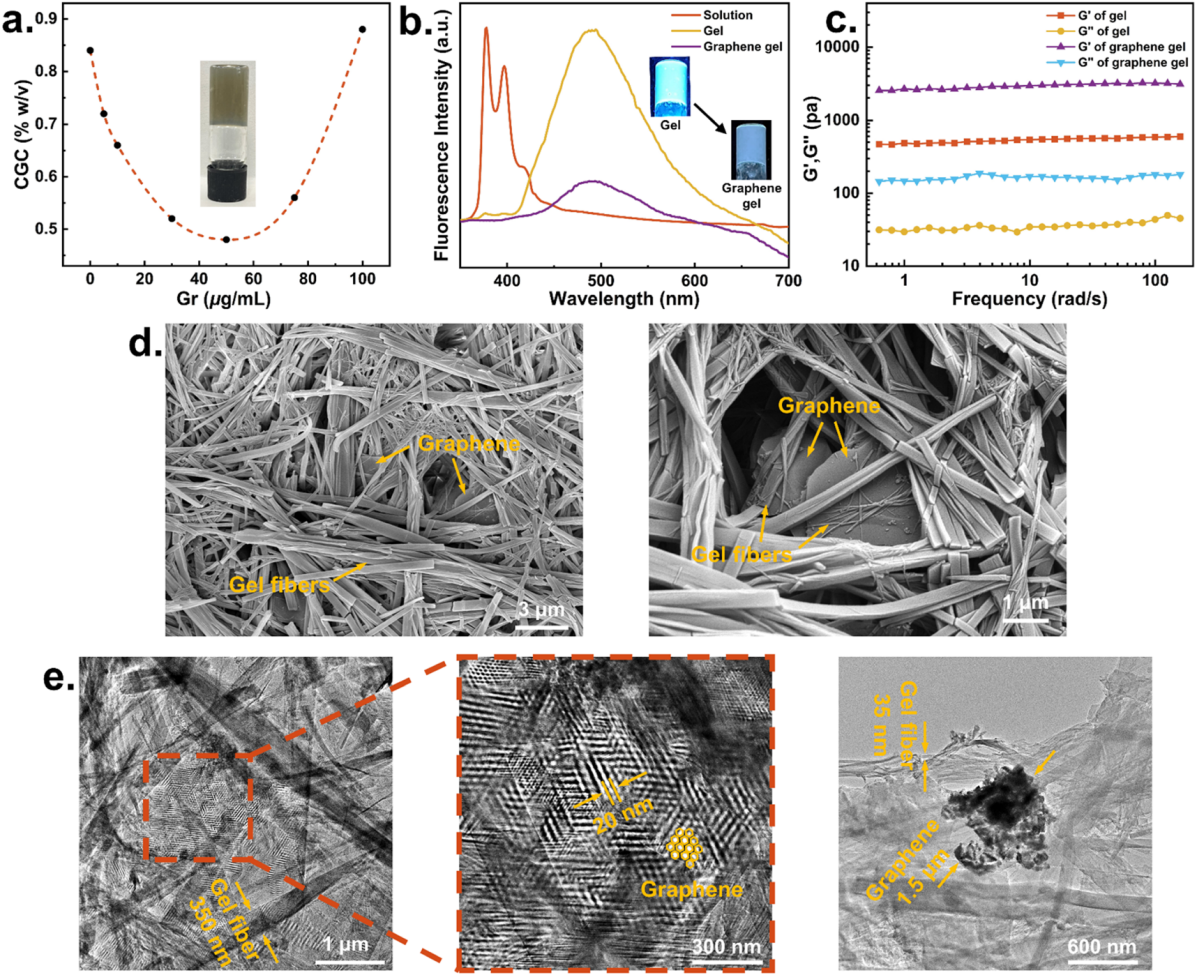
(a) CGC variation of gels at different graphene contents and photo of graphene gel at the minimum CGC; (b) fluorescence spectra of gelator solution, native gel and graphene gel in DMSO and photos of native and graphene gels under exposure to UV light at 365 nm; (c) oscillatory frequency sweeps of native and graphene gels; (d) SEM and (e) TEM images of graphene gel in DMSO.

To obtain the graphene hybrid gels, DMSO dispersions with varying graphene contents were prepared by sonication, and then gelator 2 was added and the mixture was heated to dissolve the gelator. The well-dispersed solution was sonicated during cooling to room temperature, which not only assisted the formation of the gel, but also promoted the uniform distribution of the graphene. Fig. 2a shows that the CGC of the gel initially decreases and then increases with increasing graphene content, reaching a minimum CGC of 0.48% w/v when the graphene content is 50 μg mL^−1^. This trend in the CGC of graphene gels indicates that the gelator interacts with graphene, and that the graphene significantly enhances the gelation behavior. In the absence of gels, only 3.7 μg mL^−1^ of graphene can be uniformly dispersed in pure DMSO solvent.^46^ The concentration of 50 μg mL^−1^ at the minimum CGC of the DMSO gel of 2 represents a more than tenfold enhancement in the amount of graphene incorporated into the system, resolving the drawback of low dispersion concentration of graphene in solution.

Considering the fluorescent nature of pyrene moiety, the interaction of gelator molecules in solution, native and graphene gels in DMSO was compared by fluorescence spectroscopy, shown in Fig. 2b. In solution, the gelator molecule shows the emission peaks at 378, 397 and 418 nm, which is a typical fluorescence pattern of pyrene arising from the non-aggregated pyrene-containing gelator.^47^ In native gel, the gelator experiences a self-assembly process and exhibits a strong excimer emission peak centered at 490 nm, indicating that pyrene groups of these gelator are dimerized in the gel state. The significant redshift of emission peak of native gel compared to the solution is attributed to the presence of strong π–π stacking interactions between the gelators.^48^ In graphene gel, the excimer peak is drastically quenched (7-fold reduction in intensity) compared to the native gel, proving that graphene is intercalated between the pyrene groups of gelators in supramolecular self-assembly by aromatic stacking interaction.^41,49^ The fluorescence quenching is also observed by exposing native and graphene gels to UV light. Such close interaction between graphene and pyrene groups is believed to facilitate the gelator molecule aggregation and further promote the supramolecular assembly and gelation, which explains the initial decrease in CGC of the gel with increasing graphene content.^43^

Rheology studies were performed to compare the rigidity and flow properties of native and graphene gels. Fig. 2c shows the variation of storage modulus (*G*′) and loss modulus (*G*′′) with constant strain at room temperature in frequency sweep experiments for native gel at a concentration 1.5% w/v and graphene gel containing 1.5% w/v gelator and 50 μg mL^−1^ graphene. For both native and graphene gels, the elastic behavior of the system dominates, with *G*′ being an order of magnitude larger than *G*′′, indicating a soft “solid-like” gel material. Interestingly, the incorporation of graphene increases the *G*′ of the hybrid gel by about 5-fold compared to that of the native gel, suggesting that the graphene-containing gel is more rigid than the native gel. The difference of the two moduli (Δ*G* = *G*′ − *G*′′) at frequency 0.63 rad s^−1^ shows a higher Δ*G* value for the graphene gel (2390.5 pa) than the native gel (439.1 pa), demonstrating that the elastic behavior dominates over the viscous behavior in the graphene hybrid gel.

Electron microscopy of the graphene-gel composites gives direct evidence of the intimate incorporation of graphene into the gel. SEM images show the presence of graphene nanosheets and entangled gel nanofibers in the graphene hybrid gels, with the graphene nanosheets included into the gel network. The attached gel fibers are observed on the surface of some graphene sheets, suggesting a close interaction between the gel fibers and graphene (Fig. 2c). TEM images show graphene nanosheets with a honeycomb lattice structure and black graphene flakes with a size of 1.5 μm incorporated in cross-linked gel nanofibers, further confirming that the graphene hybrid gel is a true nanocomposite system (Fig. 2d). The hexagonal lattice size of honeycomb graphene is about 20 nm, much larger than the lattice constant of 0.246 nm for single-layer graphene, which is due to the Moiré superlattice effect caused by the misaligned stacking of multilayer graphene.^50,51^ Besides, the width of nanofibers in graphene gel is 20–350 nm, which is similar to the size of native gel fibers.

### Functionalization of graphene gel

**Fig. 3:**
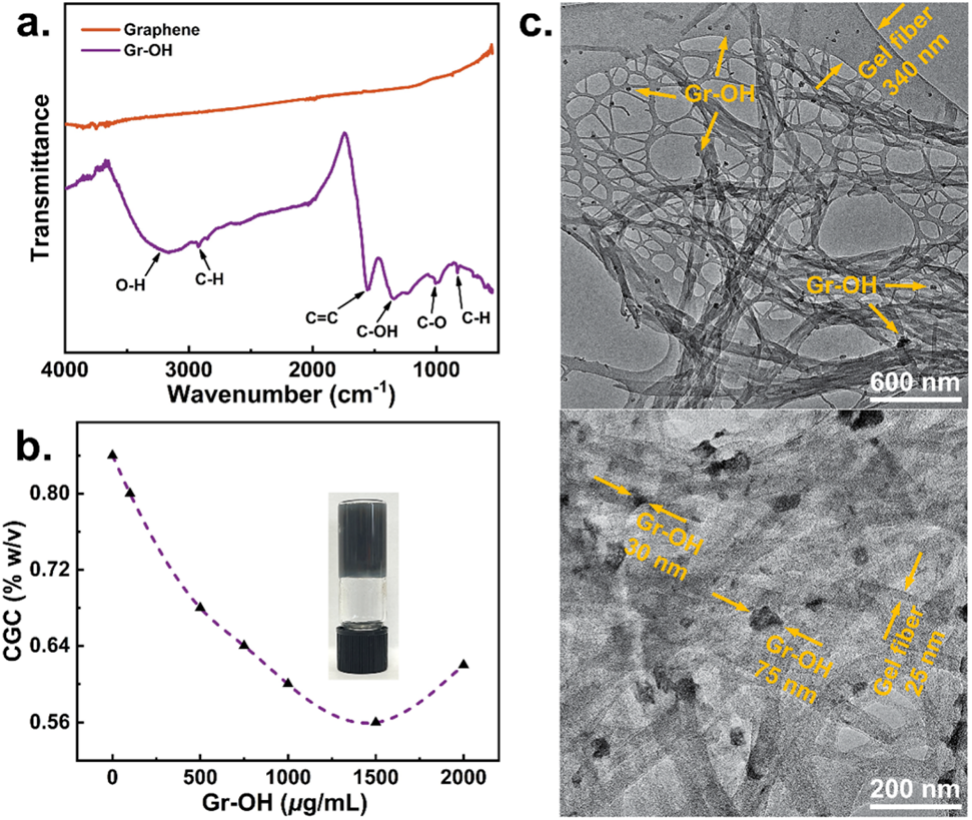
(a) FTIR spectra of graphene and Gr–OH; (b) CGC variation of gels at different Gr–OH contents and photo of Gr–OH gel at the minimum CGC; (c) TEM images of the Gr–OH gel in DMSO.

Oxygen-containing groups on graphene have the potential to interact closely with drug molecules through hydrogen bonding, allowing graphene-based gels to provide an effective nucleation surface for controlling drug crystallization. Therefore, we used thermal reduction of graphene oxide and ball milling to prepare graphene with high hydroxyl content. The surface characteristics of hydroxylated graphene (Gr–OH) nanosheets were examined by Fourier transform infrared spectroscopy (FTIR), shown in Fig. 3a. Compared to the unfunctionalized graphene, the Gr–OH exhibited strong C=C vibration from sp^2^ bonds at 1559 cm^−1^, as well as C–OH and C–O vibrations at 1342 and 1006 cm^−1^. The absorption peaks at 2924 and 831 cm^−1^ were assigned to the stretching and bending vibrations of C–H groups. The strong broad peak at 3256 cm^−1^ assigned to the OH stretching vibration suggests that a significant number of hydroxyl groups were successfully grafted onto the graphene backbone.

The properties of Gr–OH gels of 2 were characterized by CGC measurements and TEM, which indicated the incorporation of Gr–OH into the DMSO gel. Fig. 3b shows that the CGC of Gr–OH gel decreases and then increases with the increase of Gr–OH content, which is consistent with the CGC trend observed for the unfunctionalized graphene gel (Fig. 2a). The CGC of Gr–OH gel varies more gradually compared to the graphene gel, which reaches a minimum CGC of 0.56% w/v at a Gr–OH content of 1500 μg mL^−1^. After the minimum CGC, the CGC of Gr–OH gel begins to increase, indicating that more gelator is needed to interact with Gr–OH for effective gelation. TEM images display direct evidence of Gr–OH incorporation into the gel and interacting with the gelator (Fig. 3c). The hybrid gel reveals the presence of both cross-linked gel nanofibers and black Gr–OH nanosheets. The Gr–OH nanosheets attach to the surface of the entangled fibers rather than lodge in the porous pockets of the gel network, indicating that the Gr–OH nanosheets have close interaction with the gel nanofibers within the nanocomposite system.

The width of fibers in the Gr–OH gel is 25–340 nm, which is almost the same fiber size as the native and graphene gels, meaning that the incorporation of Gr–OH does not significantly change the network structure of the gel. Gr–OH is prepared by mechanical milling (see Section 2.4 of ESI†) with a size of 30–75 nm, therefore its size is much smaller than the graphene sheet, but it is still considered to serve as a heterogeneous nucleation site for drug crystallization.^5,52^ The above characterization also confirms that the designed pyrene-containing supramolecular gel can universally and easily incorporate various types of graphene into the gel system.

### Pharmaceutical crystallization in Gr–OH gels

**Fig. 4:**
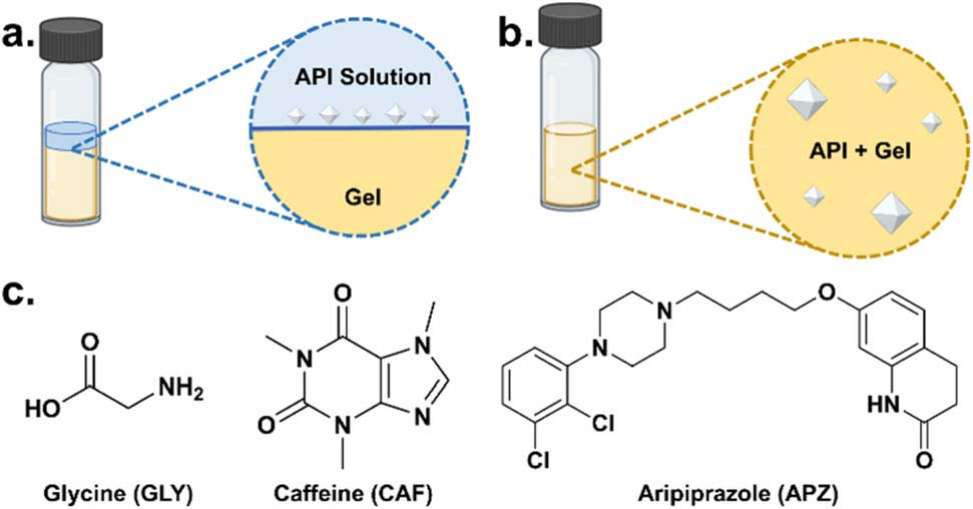
Schematic of crystallization methods of (a) gel surface crystallization and (b) gel phase crystallization; (c) three drug systems for gel crystallization.

Our previous studies have demonstrated that bis-urea supramolecular gels are formed by a scrolling mechanism in which in this case the terminal pyrene groups of gelator 2 are arranged at the outer layer of gel fibers, allowing the functionalized graphene that is incorporated between the pyrene groups to be exposed on the fiber surface and become a suitable site for API nucleation. The graphene gels were prepared in DMSO, so for API systems insoluble in DMSO, crystals can be obtained by liquid diffusion of an API solution in a different solvent at the gel surface (Fig. 4a), as previously used in cisplatin gel crystallization.^53^ For DMSO soluble APIs, crystallization can be induced in the gel phase by cooling-induced supersaturation (Fig. 4b). The incorporation of Gr–OH into the gel is expected to have an effect on the polymorphism and crystallization process of the API by interaction with the hydroxyl groups on the graphene. Therefore, three potential drug systems were selected with known extensive polymorphism and hydrogen bonding functionality as model systems for the study of crystallization process, namely glycine (GLY), caffeine (CAF), and the schizophrenia treatment aripiprazole (APZ), shown in Fig. 4c. The graphene-free native gel and Gr–OH hybrid gel were compared as crystallization matrix to conduct detailed control crystallization experiments for the three API systems to explore the template effect and the influence on crystallization process of Gr–OH in the gel (see Section 6–9 of the ESI†). The pure crystals isolated from the gel were characterized by powder X-ray diffraction (PXRD) to further identify the polymorphism by comparing with standard patterns, where crystals on the gel surface can be obtained by direct picking and crystals in the gel phase are recovered by depolymerizing the gel and filtration, as described in Section 4 of the ESI.†

**Fig. 5:**
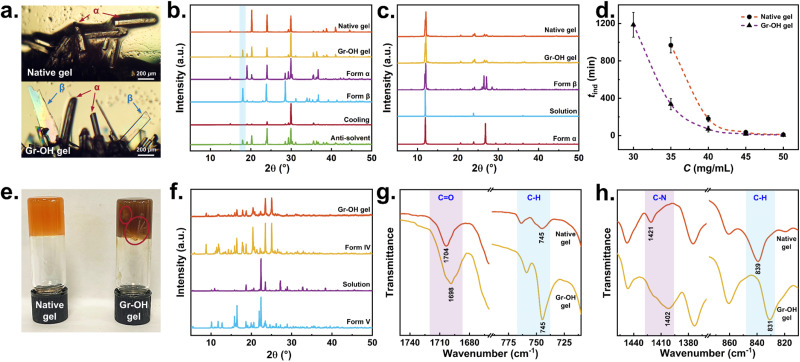
Pharmaceutical crystallization: (a) crystal morphology of GLY obtained from native gel and Gr–OH gel surface; (b) PXRD patterns of GLY by gel surface and solution crystallization; (c) PXRD patterns of CAF by gel phase and solution crystallization; (d) induction time of CAF in native gel and Gr–OH gel phase crystallization; (e) photos of APZ in native gel and Gr–OH gel phase crystallization with APZ crystals in red circles; (f) PXRD patterns of APZ in Gr–OH gel phase and solution crystallization; FTIR spectra of (g) CAF and (h) APZ in native gel and Gr–OH gel phase crystallization.

GLY is insoluble in DMSO, so an undersaturated aqueous solution of GLY was added dropwise to the surfaces of native and Gr–OH gels, respectively. At room temperature, as the water slowly diffuses into the DMSO, GLY crystallize at the interface of the gel and the solution with a low supersaturation degree, shown in Fig. 5a. The GLY crystals obtained on the native gel surface are Form α, while mixed polymorphs of Forms α and β are obtained on the Gr–OH gel surface, as identified by PXRD in Fig. 5b. Although experimenting with different drop volumes of GLY solution and Gr–OH contents in the gel, pure crystals of Form β were not obtained on the Gr–OH gel surface (see Table S1 of ESI†). This might be because Form β is the most unstable polymorph of GLY and readily transforms to Form α at room temperature.^54,55^ In addition, Fig. 5b shows the PXRD patterns of GLY crystallized from solution under the same conditions as a control. Form α is obtained from DMSO–H_2_O solution by cooling crystallization and a mixture of Forms α and β are obtained by adding GLY aqueous solution into DMSO for anti-solvent crystallization, suggesting that Form β generally occurs at high supersaturation in solution crystallization (see Tables S2 and S3 for ESI†). Previous work has reported the failure to obtain Form β at low supersaturation by conventional solution crystallization,^55^ but Form β crystallized at low supersaturation occurs on the surface of Gr–OH gel, suggesting that Gr–OH sheets in the gel can act as a template to provide specific nucleation sites for Form β to induce its crystallization.

The solubility of CAF in DMSO at room temperature is about 13 mg mL^−1^, and it was added into the native and Gr–OH gels for gel phase crystallization, respectively. CAF crystals obtained from the native and Gr–OH gels are both identified as Form β (Fig. 5c), but the crystallization timescales are different in both gels. The induction time (*t*_ind_) for crystallization of CAF in the native gel and the gel with 1000 μg mL^−1^ Gr–OH was determined, shown in Fig. 5d. At low supersaturation, CAF within Gr–OH gel shows a shorter induction time and faster nucleation rate. Specifically, when the CAF concentration (*C*) is 30 mg mL^−1^, crystals are obtained within the Gr–OH gel, while no crystals appear within the native gel. With the increase of concentration, the induction time of CAF in both gels gradually converge, when the degree of supersaturation becomes the controlling factor. In addition, solution crystallization was performed as a control experiment, which obtained the CAF crystals of Form α (see ESI Tables S4 and S5† for experimental details). Form α is a metastable polymorph containing a partially disordered structure, and Form β is the thermodynamically stable polymorph under ambient conditions.^56^ Different CAF polymorphs obtained by solution and gel phase crystallization indicate that the gel network is able to confine the convection and diffusion of CAF molecules and thus eliminate the structural disorder to obtain Form β.^53^ The shorter induction time of CAF in Gr–OH gel indicates that the presence of Gr–OH can lower the nucleation energy barrier and thus promote the nucleation of CAF.

APZ has a solubility of about 115 mg mL^−1^ in DMSO at room temperature. At low supersaturation with 150, 200 and 250 mg mL^−1^, APZ did not crystallize in either native gel or Gr–OH gel. When the concentration was increased to 300 mg mL^−1^, APZ crystals were obtained in Gr–OH gel after about 2 days at room temperature, but APZ still did not crystallize in the native gel after 7 days (Fig. 5e). Fig. 5f shows that the APZ crystals obtained from Gr–OH gel are identified as Form IV. In contrast, APZ crystals are obtained by cooling crystallization in DMSO solution without gel and are identified as Form V (see ESI Tables S6 and S7† for experimental details). APZ exists in twelve polymorphs with their transformation significantly affected by the solvent.^57^ Forms IV and V are both metastable polymorphs, and Form IV has higher a kinetic stability.^58^ Different APZ polymorphs were obtained in the solution and Gr–OH gel containing 300 mg mL^−1^ APZ under the same crystallization conditions, indicating that the Gr–OH gel environment can help the APZ molecules to overcome the solvent effect to obtain kinetically stable Form IV at the same supersaturation. No APZ crystals were obtained in the native gel, probably due to the absence of Gr–OH in the gel making it difficult for CAF molecules to nucleate with higher energy barrier, as above CAF has a longer induction time in the native gel.

For above three API systems, compared to the native gel without graphene, GLY appears as different polymorphs on the surface of Gr–OH gel, CAF in the Gr–OH gel phase has a shorter induction time, and APZ is more easily crystallized in Gr–OH gel. All results prove that Gr–OH in the gel significantly affects the polymorphic outcome, nucleation energy barrier and crystallization difficulty of APIs through specific interactions. Additionally, it should be noted that the diffraction peak intensities of experimental and standard PXRD patterns in Fig. 5 are different (probably due to changes in crystal habit and hence preferred orientation effects), but the polymorphism of APIs is primarily determined by recognizing the positions of diffraction peaks.^59^

FTIR spectroscopy was further used to monitor the interaction between Gr–OH nanosheets and API molecules in gel phase crystallization. Due to the low addition of Gr–OH and gelator in the gels, the FTIR spectra of both native and Gr–OH gels show only solvent absorption peaks but no peaks from the Gr–OH and gelator, and the FTIR spectra of both gels with addition of API show the absorption peaks of API and solvent (see Fig. S1–S3 of ESI†). Therefore, a comparison of the changes of API absorption peaks in the gel was chosen to determine the effect of Gr–OH. Fig. 5g shows the peak shift of C=O of CAF from 1704 to 1698 cm^−1^ and the change of relative peak intensity of C–H of CAF at 745 cm^−1^ in the presence of Gr–OH in the gel. Fig. 5h shows that the absorption peaks of C–N and C–H of PZA are shifted from 1421 to 1402 cm^−1^ and from 839 to 831 cm^−1^ as well as a broader peak at 1402 cm^−1^ in the presence of Gr–OH in the gel. The C=O, C–N and C–H can form strong hydrogen bonds with hydroxyl group,^60^ which demonstrates that Gr–OH in the gel can act as a nucleation site and influence the crystallization process through hydrogen bonding interactions with specific groups of API molecule.

## Conclusions

Two pyrene-containing gelators were synthesized in a high yield, and the gel formed by gelator 2 in DMSO exhibited a highly cross-linked nanofibrous structure, which was selected for subsequent graphene incorporation and pharmaceutical crystallization. Compared to the native gel, the hybrid gel containing 50 μg mL^−1^ graphene has about 40% lower CGC value and about 5-fold higher rigidity. Fluorescence study shows that graphene is intercalated between the pyrene groups of gelators by aromatic stacking interaction, and SEM and TEM visualize the close interaction of graphene sheets with gel fibers, confirming that the graphene hybrid gel is a true nanocomposite system. Graphene with high hydroxyl content was further synthesized, and CGC and TEM characterization determine that functionalized graphene also interacts well with the gel fibers and does not change the nanofiber network structure of the gel. Finally, based on the gel surface and gel phase crystallization strategies, Gr–OH in the gel state can act as a template for hydrogen bonding interactions with specific groups of API molecules, achieving the regulation of polymorphism, induction time, and nucleation difficulty during the crystallization process. This study proves that the prepared pyrene-containing gels can incorporate both pure and functionalized graphene to form hybrid systems, providing a novel pharmaceutical crystallization matrix. Further considering the easy chemical modification of graphene, the graphene gel crystallization strategy allows the incorporation of functionalized graphene designed by the API properties in the gel system and develop a generally applicable toolbox of graphene gels, promising in the accurate control of nucleation behavior and selective preparation of targeted polymorph.

## Data availability

Underlying data for this work is available at DOI: 10.15128/r1df65v7911.

## Author contributions

XZ and JWS conceived the project. YX, XZ and JWS supervised the project. QZ carried out the gel preparation, graphene preparation and pharmaceutical crystallization, MAS provided the synthesis idea of gelators, and LB carried out SEM and TEM. QZ contributed to the overall analysis and wrote the original draft, XZ and JWS contributed to the review and editing of paper.

## Conflicts of interest

There are no conflicts to declare.

## Supplementary Material

SC-016-D4SC08087D-s001
