# Supplementary material for: A tailored graphene supramolecular gel for pharmaceutical crystallization
Source: Chem Sci. 2025 Mar 24;16(17):7459–66. doi: 10.1039/d4sc08087d (PMC11949076; doi:10.1039/d4sc08087d)
Supplement: SC-016-D4SC08087D-s001 [file SC-016-D4SC08087D-s001.pdf]

Supplementary Information (SI) for Chemical Science.
This journal is © The Royal Society of Chemistry 2025

A Tailored Graphene Supramolecular Gel for Pharmaceutical Crystallization

Qi Zhang[a], Martin A. Screen[b], Leon Bowen[c], Yisheng Xu[a], Xiangyang Zhang[a,*] and Jonathan W. Steed[b,*]

[a] *State Key Laboratory of Chemical Engineering, East China University of Science and Technology, Shanghai, 200237, China.*

[b] *Department of Chemistry, Durham University, Durham, DH1 3LE, UK.*

[c] *Department of Physics, Durham University, Durham, DH1 3LE, UK.*

Corresponding author: zxydcom@ecust.edu.cn (X. Zhang), jon.steed@durham.ac.uk (J. W. Steed).

Supplementary Information

1. Materials 2
2. Synthesis 2
3. Preparation of gels 4
4. Crystallization methods 4
5. Induction time measurement 5
6. Characterization methods 5
7. Gel surface and solution crystallization of GLY 6
8. Gel phase and solution crystallization of CAF 7
9. Gel phase and solution crystallization of APZ 8
10. FTIR of gels 10
References 12

1. Materials

1,6-diisocyanatohexane, 1-pyrenemethylamine, triethylamine, 4,4′-methylenebis (2,6-diethylaniline), di-tert-butyl dicarbonate, 4-dimethylaminopyridine, magnesium sulphate, sodium hydroxide, glycine, caffeine, and tetrabutylammonium acetate were purchased from Sigma Aldrich (UK). Aripiprazole and all organic solvents were from Fisher Scientific (UK). Graphene and graphene oxide were purchased from Shenzhen Suiheng Technology Co. (China). All chemicals and reagents are of analytical purity.

2. Synthesis

2.1 Gelator 1

1,6-diisocyanatohexane (168.2 mg, 1.0 mmol) dissolved in chloroform (10 mL) was slowly added dropwise to a suspension of 1-pyrenylmethanamine hydrochloride (535.5 mg, 2.0 mmol) with slight excess of triethylamine in chloroform (40 mL). The mixture was then heated under reflux at 70°C for 18 h, cooled to room temperature, and filtered. The precipitate was then washed twice with chloroform and diethyl ether, and dried. The solid was ground to a fine white powder (479.0 mg, 0.76 mmol, 76%).

^{1}H NMR (400 MHz, DMSO-d_6) δ = 8.43-8.00 (m, 18H, Py$\underline{H}_9$), 6.46 (t, J = 5.9, 2H, PyCH$_2$N$\underline{H}$), 5.94 (t, J = 5.8, 2H, CH$_2$N$\underline{H}$), 4.95 (d, J = 5.8, 4H, PyC$\underline{H}_2$), 3.05 (q, J = 6.5, 4H, NHC$\underline{H}_2$), 1.391 (m, 4H, NHCH$_2$C$\underline{H}_2$), 1.27 (p, J = 3.5, 4H, NHCH$_2$ CH$_2$C$\underline{H}_2$). Elemental analysis for $C_{42}H_{38}N_4O_2$: Calc. (%): C 79.97, H 6.07, N 8.88; Found (%): C 79.08, H 5.87, N 8.73.

2.2 4,4′-Methylenebis (2,6-diethylphenyl isocyanate)

This compound was prepared by the previously published procedure[1]. 4,4′-Methylenebis(2,6-diethylaniline) (8.00 g, 25.8 mmol) was added to a stirred solution of di-tert-butyl dicarbonate (12.04 g, 55.2 mmol) and 4-dimethylaminopyridine (0.67 g, 5.4 mmol) in acetonitrile (100 mL) under nitrogen atmosphere. The reaction was stirred for 2 h at room temperature. 10

mL mixture of concentrated sulfuric acid and dimethylformamide (v/v = 2/3) was quickly added and the reaction left to stir for a further 5 min. The reaction mixture was added to an equal amount of water and extracted into hexane. The hexane layers were combined, dried with magnesium sulphate and evaporated under vacuum. Dichloromethane was added to the resulting slurry and the filtrate was slowly evaporated to give 4,4′-Methylenebis (2,6-diethylphenyl isocyanate) as a white solid (7.68 g, 21.2 mmol, 82%).

^{1}H NMR (400 MHz, DMSO-d_6) δ = 6.99 (s, 4H, Ph$\underline{H}_2$), 3.83 (s, 2H, PhC$\underline{H}_2$Ph), 2.59 (q, J = 7.5 Hz, 8H, C$\underline{H}_2$CH$_3$), 1.14 (t, J = 7.5 Hz, 12H, CH$_2$C$\underline{H}_3$). Elemental analysis for $C_{23}H_{26}N_2O_2$: Calc. (%): C 76.21, H 7.23, N 7.73; Found (%): C 75.88, H 7.26, N 7.63.

2.3 Gelator 2

4,4′-Methylenebis(2,6-diethylphenyl isocyanate) (362.2 mg, 1.0 mmol) dissolved in chloroform (10 mL) was added dropwise to a suspension of 1-pyrenylmethanamine hydrochloride (535.5 mg, 2.0 mmol) with slight excess of triethylamine in chloroform (40 mL). The mixture was then heated under reflux at 70°C for 18 h, cooled to room temperature, and filtered. The precipitate was then washed twice with chloroform and diethyl ether, and dried. The solid was ground as a fine milky yellow powder (692.5 mg, 0.84 mmol, 84%).

^{1}H NMR (400 MHz, DMSO-d_6) δ = 8.47-8.04 (m, 18H, Py$\underline{H}_9$), 7.42 (s, 2H, PhN$\underline{H}$), 6.95 (s, 4H, Ph$\underline{H}_2$), 6.73 (s, 2H, PyCH$_2$N$\underline{H}$), 5.02 (s, 4H, PyC$\underline{H}_2$), 3.82 (s, 2H, PhC$\underline{H}_2$Ph), 2.52 (m, 8H, PhC$\underline{H}_2$), 1.07 (t, J = 7.6, 12H, PhCH$_2$C$\underline{H}_3$). Elemental analysis for $C_{57}H_{52}N_4O_2$: Calc. (%): C 82.98, H 6.35, N 6.79; Found (%): C 82.40, H 6.30, N 6.71.

2.4 Hydroxylated graphene (Gr-OH)

Gr-OH was synthesized with a similar method as in literature[2]. To obtain graphene with high hydroxyl content, graphene oxide was first performed thermal reduction at 400°C. The thermally reduced graphene oxide was then

reacted with sodium hydroxide (m/m = 1/40) by ball milling for 10 h. The solid was washed to neutrality with deionized water, filtered and freezing-dried to obtain Gr-OH. FTIR was used to determine the oxygen-containing groups on Gr-OH.

3. Preparation of gels

3.1 Gel

The required amount of gelator and organic solvent were added in a screw-top glass vial and slowly heated to dissolve with a heat gun. The solution was then slowly cooled and sonicated for about 10 seconds to room temperature. The gelation was checked by the “stable-to-inversion” test on the aggregated material in the vial.

3.2 Graphene gel

A certain amount of graphene (or Gr-OH) was added to the organic solvent and fully sonicated to obtain graphene (or Gr-OH) solutions with different dispersions. The required amount of gelator was added in the dispersion and slowly heated. Once the gelator was dissolved, sonication was performed to ensure uniform dispersion of graphene (or Gr-OH) until gelation occurred.

4. Crystallization methods

4.1 Gel surface crystallization

Gel surface crystallization was used when API was insoluble in the organic solvent of gel (antisolvent). At room temperature, by adding dropwise an unsaturated solution of a certain amount of API-good solvent to the surface of gel, the good solvent diffused into the gel, causing the API to reach supersaturation and crystallize on the interface. The crystals can be taken directly from the gel surface.

4.2 Gel phase crystallization

When API was soluble in the organic solvent of gel, it was added to the solvent with gelator and then was prepared as the API-gel. API was

supersaturated at room temperature allowing it to crystallize in the gel phase. The crystals in the gel phase can be recovered by adding tetrabutylammonium acetate to depolymerize the gel and then filtration, following the recovery method in our previous papers.[3, 4]

5. Induction time measurement

To compare the effects of gel and Gr-OH gel on the nucleation process of CAF, the induction time (t_{ind}) was determined. In gel phase crystallization, different concentrations of CAF were added to gel and Gr-OH gel, respectively. Once the gel was formed at room temperature (20°C), timing was started. Due to the translucent nature of the gel, the time of obtaining crystals can be determined by direct observation method. Considering the randomness of the nucleation process, each condition was performed 5 times in parallel and the average value was taken as the value of the t_{ind}.

6. Characterization methods

6.1 Scanning electron microscopy (SEM)

Gel samples were placed on silicon wafers and dried at room temperature. The samples were sputter-coated with 2 nm of platinum and imaged by a FEI Helios NanoLab DualBeam microscope in immersion mode with typical beam settings.

6.2 Transmission electron microscopy (TEM)

Gel samples were placed on a 300 mesh carbon-coated Cu grid and dried at room temperature. TEM images were obtained using a high-resolution mode using a JEOL 2100F FEG.

6.3 Fluorescence spectroscopy

The gelator solution was prepared by dissolving 0.01% gelator **2** in DMSO and transferred to a quartz cell. The native and graphene gels were both heated to dissolve the gelator in a glass vial and then transferred to a quartz dish to form the gel as described in the gel preparation method above, with

native gel containing 0.85% w/v gelator **2** and graphene gel containing 0.48% w/v gelator **2** and 50 *μ*g/mL graphene. The fluorescence emission spectra were collected by a Shimadzu RF-6000 at 335 nm excitation wavelength from 350 nm to 700 nm. The emission and excitation slit widths were both 5 nm, and the path length of quartz cell was 1 cm.

6.4 Rheology

Rheology experiments were performed using a Kinexus Pro+ on a rough Peltier plate with 25 mm geometry and 1.0 mm plate gap at room temperature. Frequency sweep experiments were performed with a constant applied stress of 1 Pa.

6.5 Fourier transform infrared (FTIR) spectroscopy

FTIR spectrum of Gr-OH was measured on a Perki Elmer Spectrum 100 instrument using direct compression method in the wavelength range of 4000-550 cm^{-1}.

6.6 Polarizing optical microscopy (POM)

The morphology of crystals was determined by POM with OLYMPUS SZX12.

6.7 Powder X-ray diffraction (PXRD)

The polymorph of API crystals was identified by PXRD with Bruker D8 Advance. The sample was placed in diffractometer with Cu Kα radiation (λ=1.54 Å), collecting the pattern in the range of 5-50° within 10 min.

The polymorphism of the obtained crystals was identified by comparing experimental and standard PXRD patterns. The standard PXRD patterns were obtained by simulating the single crystal structures from the Cambridge Structure Data (CSD) by Mercury software, with Refcodes GLYCIN03[5] and GLYCIN[6] for Forms α and β of GLY, respectively, Refcodes NIWFEE02[7] and NIWFEE03[8] for Forms α and β of CAF, respectively, and Refcodes MELFIT04 and MELFIT05[9] for Forms IV and V of APZ, respectively.

7. Gel surface and solution crystallization of GLY

In gel surface crystallization experiments, 100 mg/mL GLY-water solution was added dropwise to the surfaces of 1% w/v gel and Gr-OH gel at room temperature, respectively. The drop volumes of GLY solution were 100, 200 and 500 μL, and the contents of Gr-OH in 0.5 mL of DMSO-gel were 1000 and 1500 μg/mL, respectively. Form α was obtained on the gel surface, while the Gr-OH gel surface tended to crystallize Forms α and β, as shown in Table S1.

Table S1. Polymorphism of GLY by gel surface crystallization.

NO.	Content of Gr-OH (μg/mL)	Volume of GLY solution (μL)	Form
1	0	100	α
2	0	200	α
3	0	500	α
4	1000	100	α + β
5	1000	200	α + β
6	1000	500	α + β
7	1500	100	α + β
8	1500	200	α + β
9	1500	500	α + β

In cooling crystallization experiments, 100, 200 and 500 μL of water were added to 0.5 mL of DMSO, then GLY was added to the binary solvent and heated to obtain a supersaturated solution of 100 mg/mL, and the crystals were obtained by standing at room temperature, as shown in Table S2.

Table S2. Cooling crystallization of GLY in H_2O-DMSO mixed solvents.

NO.	GLY (mg/mL)	H_2O (μL)	DMSO (mL)	Form
1	100	100	0.5	α
2	100	200	0.5	α
3	100	500	0.5	α

In anti-solvent crystallization experiments, 100 mg/mL GLY-water solution was prepared and added dropwise to 0.5 mL of DMSO in volumes of 100, 200 and 500 μL at room temperature to rapidly obtain GLY crystals, as shown in Table S3.

Table S3. Anti-solvent crystallization of GLY-water solution.

NO.	GLY (mg/mL)	Addition volume (μL)	DMSO (mL)	Form
1	100	100	0.5	α + β
2	100	200	0.5	α + β
3	100	500	0.5	α + β

8. Gel phase and solution crystallization of CAF

In gel phase crystallization experiments of CAF, 1% w/v gelator **2** was added in 0.5 mL DMSO. The content of Gr-OH in gel was 1000 μg/mL, and the concentrations of CAF were 30, 35, 40, 45, and 50 mg/mL, respectively. All CAF crystals obtained were Form β, as shown in Table S4.

Table S4. Gel phase crystallization of CAF.

NO.	Content of Gr-OH (μg/mL)	CAF (*m*g/mL)	Form
1	0	30	None
2	0	35	β
3	0	40	β
4	0	45	β
5	0	50	β
6	1000	30	β
7	1000	35	β
8	1000	40	β
9	1000	45	β
10	1000	50	β

In solution crystallization, CAF was added to 0.5 mL of DMSO and heated to obtain supersaturated solutions of 30, 35, 40, 45, and 50 mg/mL, respectively, and the crystals were obtained by standing at room temperature, as shown in Table S5.

Table S5. Solution crystallization of CAF.

NO.	CAF (*m*g/mL)	Form
1	30	None
2	35	α
3	40	α
4	45	α
5	50	α

9. Gel phase and solution crystallization of APZ

In gel phase crystallization, 1% w/v gelator **2** was added in 0.5 mL DMSO. The content of Gr-OH in gel was 1000 *μ*g/mL, and the concentrations of APZ were 150, 200, 250, and 300 mg/mL, respectively, with the results shown in Table S6.

Table S6. Gel phase crystallization of APZ.

NO.	Content of Gr-OH (*μ*g/mL)	APZ (*m*g/mL)	Crystal
1	0	150	None
2	0	200	None
3	0	250	None
4	0	300	None
5	1000	150	None
6	1000	200	None
7	1000	250	None
8	1000	300	Form IV

In solution crystallization, APZ was added to 0.5 mL of DMSO and heated to obtain supersaturated solutions of 150, 200, 250, and 300 mg/mL, respectively, and the crystals of Form V were obtained by standing at room temperature, as shown in Table S7.

Table S7. Solution crystallization of APZ

NO.	CAF (*m*g/mL)	Form
1	150	V
2	200	V
3	250	V
4	300	V

10. FTIR of gels

In FTIR characterization, wet gels were directly placed on the instrument for testing. Due to the low relative amount of gelator and Gr-OH in the gels, only the absorption peaks of the solvent DMSO were detected, as shown in Fig. S1. Gel and Gr-OH gel containing supersaturated CAF and APZ were measured by FTIR, respectively, displaying the absorption peaks of API and DMSO, as shown in Figs. S2 and S3.

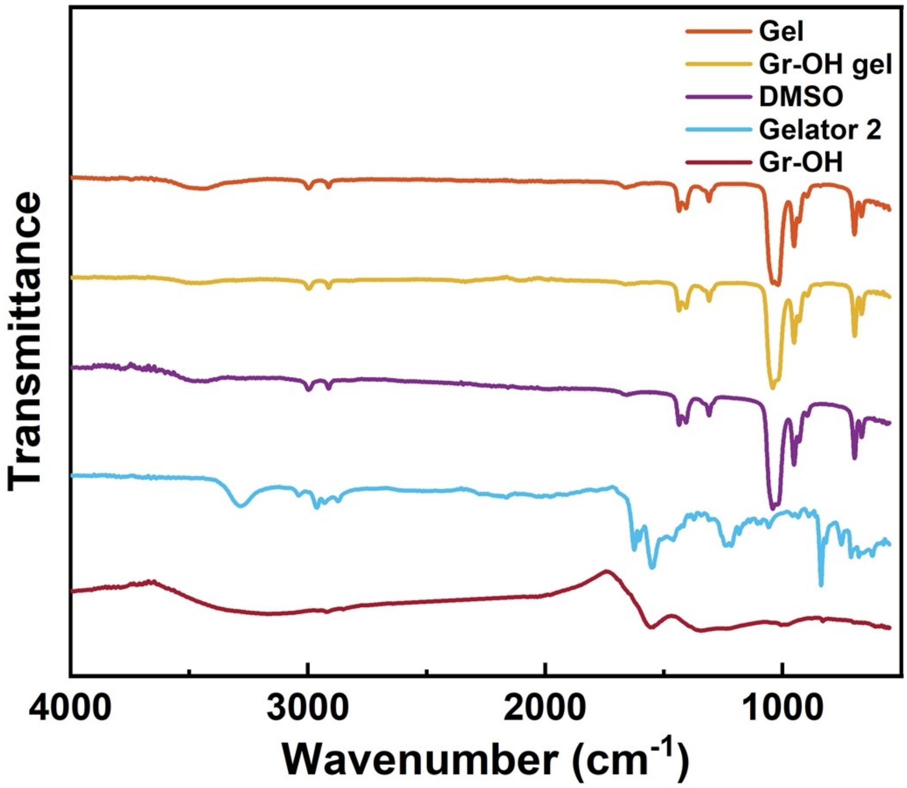

Fig. S1. FTIR spectra of gel and Gr-OH and their components.

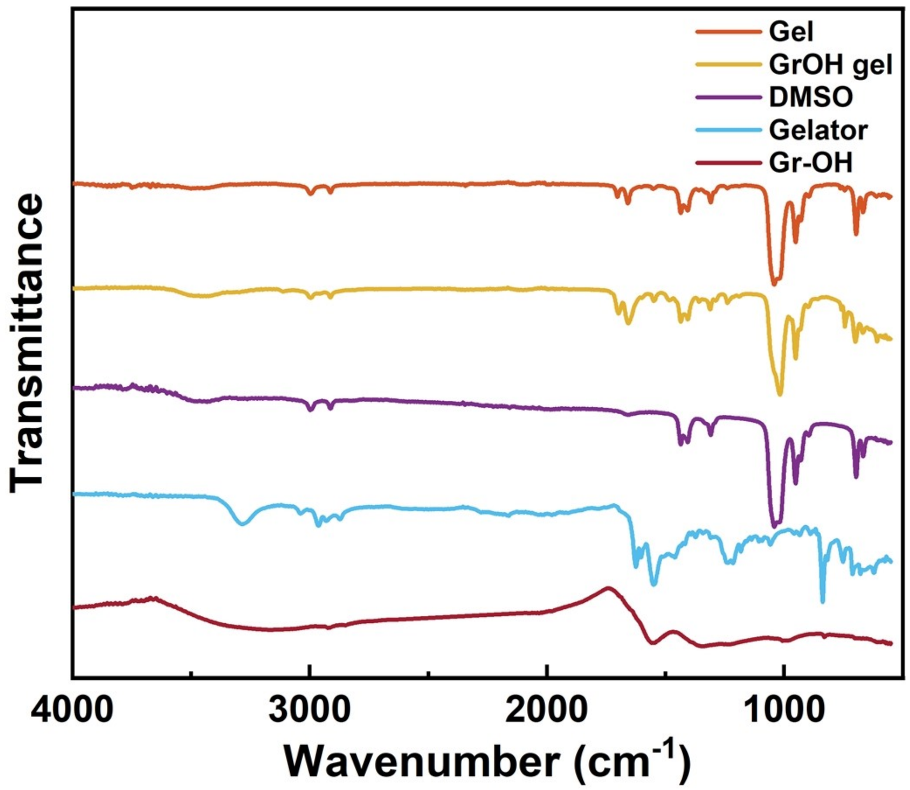

Fig. S2. FTIR spectra of CAF in gel and Gr-OH gel phase crystallization.

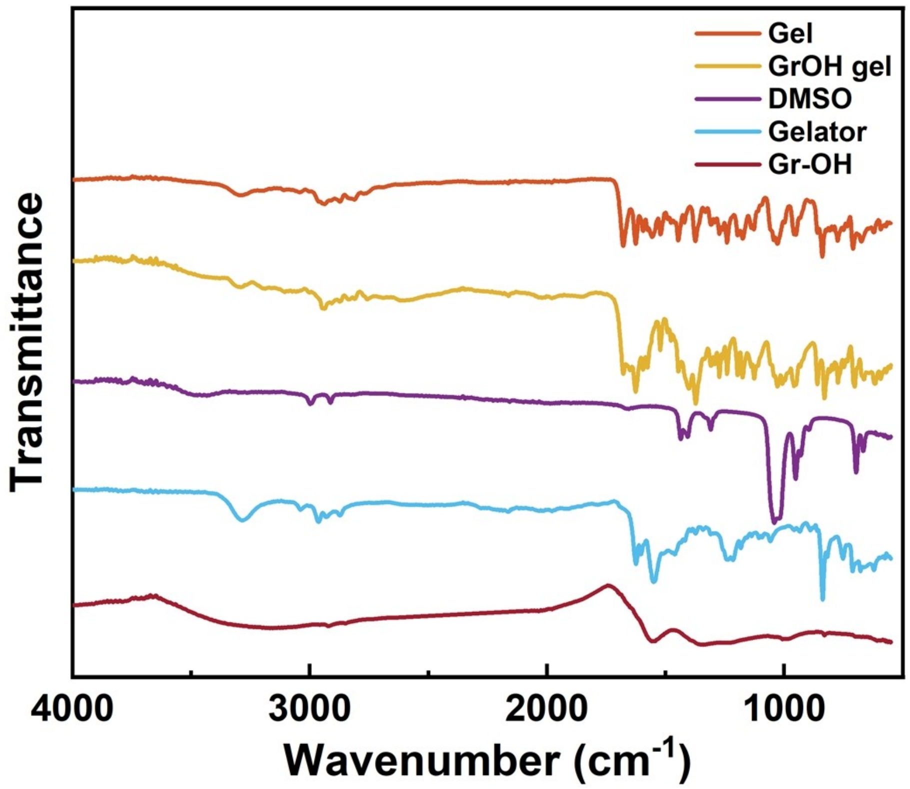

Fig. S3. FTIR spectra of APZ in gel and Gr-OH gel phase crystallization.

References

1. J. L. Andrews, S. R. Kennedy, D. S. Yufit, J. F. McCabe and J. W. Steed, *Cryst. Growth. Des.*, 2022, **22**, 6775-6785.
2. S. Yang, W. Jia, Y. Wang, W. Zhang and X. Yuan, *ACS Omega*, 2021, **6**, 30465-30477.
3. J. A. Foster, M.O. M. Piepenbrock, G. O. Lloyd, N. Clarke, J. A. K. Howard and J. W. Steed, *Nature Chem.*, 2010, **2**, 1037-1043.
4. J. A. Foster, K. K. Damodaran, A. Maurin, G. M. Day, H. P. Thompson, G. J. Cameron, J. C. Bernal and J. W. Steed, *Chem. Sci.*, 2017, **8**, 78-84.
5. P. G. Jönsson and Å. Kvick, *Acta Crystallogr. B*, 1972, **28**,1827-1833.
6. Y. Iitaka, *Acta Crystallogr.*, 1960, **13**, 35-45.
7. P. Derollez, N. T. Correia, F. Danède, F. Capet, F. Affouard, J. Lefebvre and M. Descamps, *Acta Crystallogr. B*, 2005, **61**, 329-334
8. C. W. Lehmann and F. Stowasser, *Chem. – Eur. J.*, 2007, **13**, 2908-2911.
9. D. E. Braun, T. Gelbrich, V. Kahlenberg, R. Tessadri, J. Wieser and U. J. Griesser, *J. Pharm. Sci.*, 2009, **98**, 2010-2026.
